# Mbd2 enables tumourigenesis within the intestine while preventing tumour‐promoting inflammation

**DOI:** 10.1002/path.5074

**Published:** 2018-05-16

**Authors:** Stephanie May, Heather Owen, Toby J Phesse, Kirsty R Greenow, Gareth‐Rhys Jones, Adam Blackwood, Peter C Cook, Christopher Towers, Awen M Gallimore, Geraint T Williams, Michael Stürzl, Nathalie Britzen‐Laurent, Owen J Sansom, Andrew S MacDonald, Adrian P Bird, Alan R Clarke, Lee Parry

**Affiliations:** ^1^ European Cancer Stem Cell Research Institute Cardiff University, School of Biosciences Cardiff UK; ^2^ Wellcome Trust Centre for Cell Biology University of Edinburgh, *Michael Swann Building* Edinburgh UK; ^3^ Manchester Collaborative Centre for Inflammation Research Manchester UK; ^4^ Cardiff Institute of Infection and Immunity, *Henry Wellcome Building* Cardiff UK; ^5^ Institute of Cancer and Genetics Cardiff University School of Medicine Cardiff UK; ^6^ Division of Molecular and Experimental Surgery, Department of Surgery Friedrich‐Alexander‐Universität (FAU) Erlangen‐Nürnberg and Universitätsklinikum Erlangen Erlangen Germany; ^7^ Cancer Research UK Beatson Institute Glasgow UK

**Keywords:** colon cancer, DSS colitis, inflammation, epigenetics

## Abstract

Epigenetic regulation plays a key role in the link between inflammation and cancer. Here we examine Mbd2, which mediates epigenetic transcriptional silencing by binding to methylated DNA. In separate studies the Mbd2 ^−/−^ mouse has been shown (1) to be resistant to intestinal tumourigenesis and (2) to have an enhanced inflammatory/immune response, observations that are inconsistent with the links between inflammation and cancer. To clarify its role in tumourigenesis and inflammation, we used constitutive and conditional models of Mbd2 deletion to explore its epithelial and non‐epithelial roles in the intestine. Using a conditional model, we found that suppression of intestinal tumourigenesis is due primarily to the absence of Mbd2 within the epithelia. Next, we demonstrated, using the DSS colitis model, that non‐epithelial roles of Mbd2 are key in preventing the transition from acute to tumour‐promoting chronic inflammation. Combining models revealed that prior to inflammation the altered Mbd2 ^−/−^ immune response plays a role in intestinal tumour suppression. However, following inflammation the intestine converts from tumour suppressive to tumour promoting. To summarise, in the intestine the normal function of Mbd2 is exploited by cancer cells to enable tumourigenesis, while in the immune system it plays a key role in preventing tumour‐enabling inflammation. Which role is dominant depends on the inflammation status of the intestine. As environmental interactions within the intestine can alter DNA methylation patterns, we propose that Mbd2 plays a key role in determining whether these interactions are anti‐ or pro‐tumourigenic and this makes it a useful new epigenetic model for inflammation‐associated carcinogenesis. © 2018 The Authors. *The Journal of Pathology* published by John Wiley & Sons Ltd on behalf of Pathological Society of Great Britain and Ireland.

## Introduction

Epigenetic regulation of the genome plays a major role in human health and disease 1. DNA methylation, via addition of a methyl group to the fifth carbon of cytosine in a CpG dinucleotide 2, is a fundamental epigenetic modification. As DNA methylation can be influenced by the environment, it plays a large role in the biology of diseases with a strong environmental component, such as the intestine 3, 4. DNA methylation is synonymous with transcriptional silencing as it can either inhibit binding of transcription regulators, such as c‐Myc 5, or recruit co‐repressor complexes that trigger the formation of repressive chromatin 6. These co‐repressor complexes are recruited by proteins which bind directly to methylated DNA to mediate a cell's complex multi‐layered transcriptional programme. These methyl‐binding proteins act as ‘master controllers’ by mediating the effects of DNA methylation; thus, they can regulate many genes simultaneously, including aberrantly methylated genes that lead to disease 4. One of these proteins, methyl binding domain protein 2 (Mbd2), has been shown to play crucial roles in various biological processes and diseases 4, 7. The importance of its role has been highlighted using mouse models, where its deficiency has been shown to influence intestinal inflammatory responses 8, 9, 10, 11, 12, 13 and epithelial cell biology 14, 15, 16. These multiple roles potentially make it a unifying player in the myriad of biological processes that contribute to the initiation and development of intestinal cancer.

Using the *Apc* ^*+/min*^ model 17, we have previously demonstrated that the *Mbd2* ^*−/−*^ mouse is resistant to Wnt‐driven tumourigenesis 15, 16 – the most common type of human colorectal cancer (CRC). Further, we demonstrated that *Mbd2* deficiency in a Wnt‐activated intestine reduces the expression of Wnt target genes, including *c‐Myc* 15. Potentially, this attenuation of tumourigenesis is due to a lack of *Mbd2‐*dependent silencing of tumour suppressor genes; however, the mechanism of *Apc* ^*+/min*^ *Mbd2* ^*−/−*^ tumour suppression remains unknown. During the course of these studies, we reported that *Mbd2* deficiency in Wnt‐activated intestinal epithelia relieved silencing of genes associated with immune responses 15. This is in accordance with previously reported roles for *Mbd2* in guiding cells down the different epigenetically regulated T‐cell lineages 8, 9, 10, 11, 12, 13. This presents within the intestine of the *Mbd2* ^*−/−*^ mouse as an excessive type 1 response upon immune challenge 8, characterised by an increase in the expression of the pro‐inflammatory cytokine interferon gamma (Ifng). Evidence from mice and humans suggests an anti‐tumourigenic role for Ifng in colorectal cancer (CRC), as a loss of type 1 cytokines accompanies the adenoma–carcinoma sequence in the colorectum 18; intestinal tumourigenesis is promoted in mice deficient for *Ifng* or its receptors 19; and Th1 cytokines lead to cancer senescence 20. This role is due, at least in part, to Ifng*‐*mediated *c‐Myc* inhibition 21, 22 and increased expression of the HLA‐DR antigen 21 – features we previously reported in the Wnt‐activated *Mbd2‐*deficient intestine 21. However, in its inflammatory role, Ifng is causally involved in inflammatory bowel diseases, where chronic inflammation drives cellular and molecular inflammatory mechanisms that underlie tumour initiation 23, 24. Evidence from other tissues indicates that the tussle between the anti‐ and pro‐tumourigenic functions of Ifng seems to be dependent on the contexts of tumour specificity, microenvironmental factors, and signalling intensity 25.

Here we examine how the increased inflammatory response and tumour suppression phenotype interact in the *Mbd2‐*deficient mouse intestine. Our findings highlight separate roles for *Mbd2* in controlling intestinal inflammation and enabling epithelial tumourigenesis.

## Materials and methods

### Animal models

All animal procedures were conducted in accordance with institutional animal care guidelines and UK Home Office regulations. In brief, mice were maintained in a specific pathogen‐free (SPF) barrier facility in conventional open top cages on Eco‐Pure Chips 6 Premium bedding (Datesand, Manchester, UK) under a 12 h light cycle, with IPS 5008 diet (Labdiet‐IPS Ltd, London, UK) provided for nutritional support. To enrich the environment, irradiated sunflower seeds (at weaning only), Techniplast mouse houses (Techniplast, Leicester, UK), and small chewsticks (Labdiet‐IPS Ltd) were provided. All mice were from a mixed background and were homozygous with respect to the C57Bl/6 Pla2g2a (also called Mom‐1) allele. Experimental animals were between 10 and 15 weeks old, with siblings used as controls. The alleles for *Ah‐cre* 26, *Apc* ^*+/min*^, *Ifng* ^*−/−*^ 27, *Lgr5creER* ^*T2*^ 28, *Mbd2* ^*−/−*^ 9, *Mbd2* ^*flx/flx*^ 13, and *vil‐creER* ^*T2*^ 29 have been described previously. Induction of the *Ah‐cre* transgene was performed by administering three intraperitoneal (i.p.) injections of β‐naphthoflavone (BNF; Sigma, Gillingham, Dorset, UK) at 80 mg/kg in a 24 h period. Induction of the *Lgr5creER* ^T2^ and *vil‐creER* ^*T2*^ transgenes was achieved by administering a single injection of tamoxifen (TAM; 80 mg/kg i.p.; Sigma) for four consecutive days. For induction of colitis (acute inflammatory insult), mice were given dextran sodium sulphate (MW 36 000–50 000; MP Biomedicals, Fisher Scientific, Loughborough, UK) *ad libitum* in drinking water at the concentration (w/v) and duration stated. Colitis disease severity was measured according to published protocols 30. For survival analysis, mice were harvested at either a specific time point or a humane endpoint when mice displayed phenotypes indicative of acute colitis (weight loss and diarrhoea) or tumour burden (pale feet, bloating, prolapse or piloerection). Tumour burden was measured at point of death by removing the entire intestine and mounting *en face* in methacarn fixative (4:2:1 methanol, chloroform, and glacial acetic acid) to determine the number of macroscopic lesions and their size.

### Reverse transcription–quantitative PCR (RT‐qPCR) analyses

The following methods were performed according to the manufacturer's instructions unless otherwise stated. For analysis of gene expression in the intestine, three to five mice from each control and experimental group were harvested. RNA was extracted either from a 0.5 cm portion of the whole large intestine taken ∼1 cm distally from the caecum or from crypt epithelia extracted from the whole large intestine; samples were stored at −80°C in RNAlater (Sigma, Dorset, UK) 31. Total RNA was extracted using the RNeasy kit (Qiagen, Manchester, UK) and DNase‐treated using the Turbo DNase kit (Fisher Scientific). Complimentary DNA (cDNA) was reverse transcribed from 1 μg of RNA using random hexamers (Promega, Southampton, UK) and the Superscript III kits (Fisher Scientific, Loughborough, UK). For relative quantitation, all samples were run in duplicate on the StepOnePlus PCR machine using Fast SYBR Green master mix (Applied Biosystems, Oxford, UK) or Taqman Universal Mastermix II (Fisher Scientific). The threshold cycle (Ct) values of each gene analysed were normalised to a reference gene. For expression analysis, Ct values were normalised against the *Actb* gene (mouse) or *RPL37A* (human). Oligonucleotide sequences used for relative quantification are available upon request. Differences between groups were assessed using the 2^−ΔΔCT^ method 32. Two‐tailed Mann–Whitney *U* (MW) tests were performed on the ΔCt values and differences with *P* values less than 0.05 were considered significant 33.

### Reporter visualization, immunohistochemistry (IHC), and cellular analysis

For IHC, tissue was fixed in ice‐cold 10% neutral sodium phosphate‐buffered formalin (Sigma) and processed into wax blocks by conventional means. Section were cut at 5 μm thickness, dewaxed, and rehydrated into PBS. Staining was performed using the Envision+ mouse or rabbit kit (Dako, Agilent Ltd, Stockport, UK) according to the manufacturer's instructions. To identify cells which had lost *Apc*, we used nuclear β‐catenin as a surrogate marker, using a mouse monoclonal anti‐β‐catenin antibody (Cat No 610154; BD Biosciences, Wokingham, UK) at 1:200. For Paneth cell detection, we used a rabbit polyclonal anti‐lysozyme antibody (Cat No RB‐372; Neomarkers/Labvision, Fisher Scientific) at 1:200. CD4 cells were stained with a mouse anti‐CD4 antibody (Clone 4sm95; eBioscience, Fisher Scientific; 1/50). To visualize mucin and goblet cells, slides were stained with Alcian Blue. The cells between the base of the crypt and the junction with the villus were designated as the proliferative zone. Cellular analysis was performed on more than 25 whole crypts from at least three mice of each genotype. Slides were scanned for analysis using the Axioscan Z1 slide scanner (Zeiss, Cambridge, UK) and images were excised from scans using the Zeiss Axioscan Zen software.

### T‐cell analysis

To label the Th1 and Th2 cytokines in serum, we used a Cytometric Bead Array Mouse Th1/Th2/Th17 Cytokine kit (BD Biosciences) following the manufacturer's instructions and using a FACS Canto II (BD Biosciences). To characterize the immune cell populations, single cell suspensions of lymphocytes were prepared from large intestine lamina propria 34, 35. Lymphocytes were stimulated for 4 h using ionomycin, phorbol myristate acetate (Sigma), and 1 μg/ml Golgistop (Sigma). Prior to flow cytometry, lymphocytes were stained with fluorescent conjugated antibodies against CD4 (RM‐4‐5, 1/800), Ifng (XMG1.2, 1/200), IL‐13 (eBIO 13a, 1/200), TCRa/b (MR5.2, 1/200) (all Fisher Scientific), CD45 (30‐F11, 1/200), CD8 (53‐6.7, 1/200), IL‐4 (11B11, 1/200) (all Biolegend, London, UK), IL‐17 (TC11‐18H10.1, 1/100) and TNF (MP6‐XT22, 1/200) (both BD Biosciences), and a live/dead marker. Data were analysed using FCAP Array v3.0 (BD Biosciences) and FlowJo (FlowJo LLC, Ashland, OR, USA) software. Statistical analysis was performed using three to five animals per genotype and three independent experiments. Significance was calculated using a three‐way full factorial fit model and a joint F‐test to assess the effects of genotype, treatment, and experiment day on the cytokine response 36.

### Statistical analysis

All preclinical data were evaluated with GraphPad Prism software, version 7.02 (GraphPad, La Jolla, CA, USA). When two variables were compared, a two‐tailed Mann–Whitney test was performed. Survival data were analysed using the Kaplan–Meier test. The relationship between genotype and phenotype was assessed using Fisher's exact test. If not indicated otherwise, the statistical mean is presented and error bars represent SEM. On graphs, *P* values are indicated as follows: *p < 0.05; **p < 0.01; ***p <0 .001.

## Results

### Intestinal epithelial loss of Mbd2 is sufficient to suppress tumourigenesis

**Figure 1:**
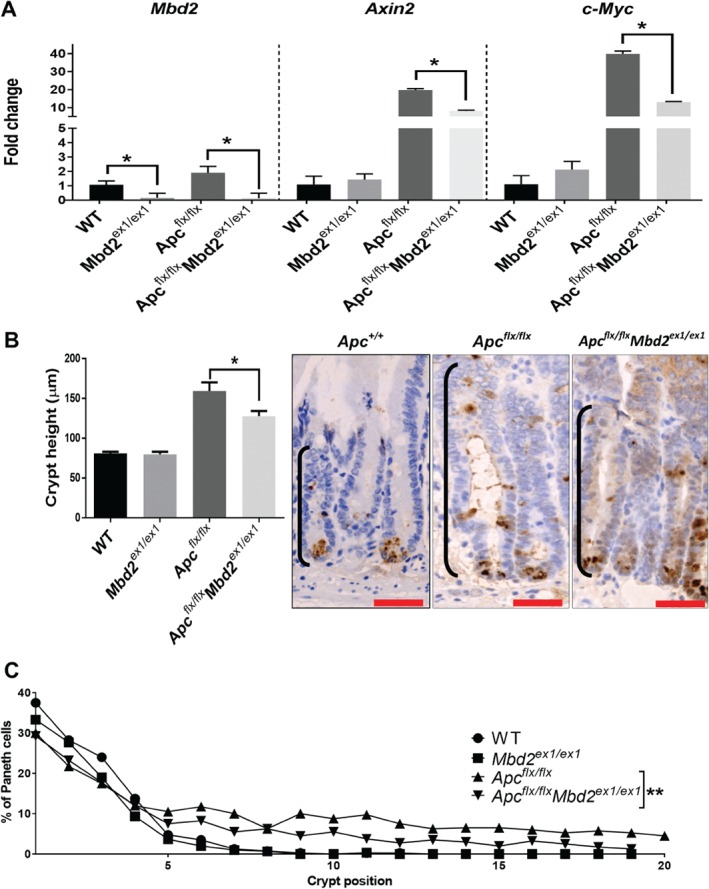
Epithelial loss of Mbd2 in the intestine attenuates the phenotype associated with Ah‐creApc deletion. (A) RT‐qPCR gene expression data indicating that Mbd2 loss suppresses the expression of Wnt target genes following Apc deletion (N = 4–6). (B) Quantification of crypt size (left panel) indicating reduction in the size of the proliferative zone and representative images of the proliferative zone and Paneth cells (brown; right panel). (C) Cumulative frequency curve of Paneth cell localisation within the intestinal crypt, indicating partial rescue of positioning in the Ah‐creApc ^flx/flx^ Mbd2 ^ex1/ex1^ intestine compared with Ah‐creApc ^flx/flx^.

**Figure 2:**
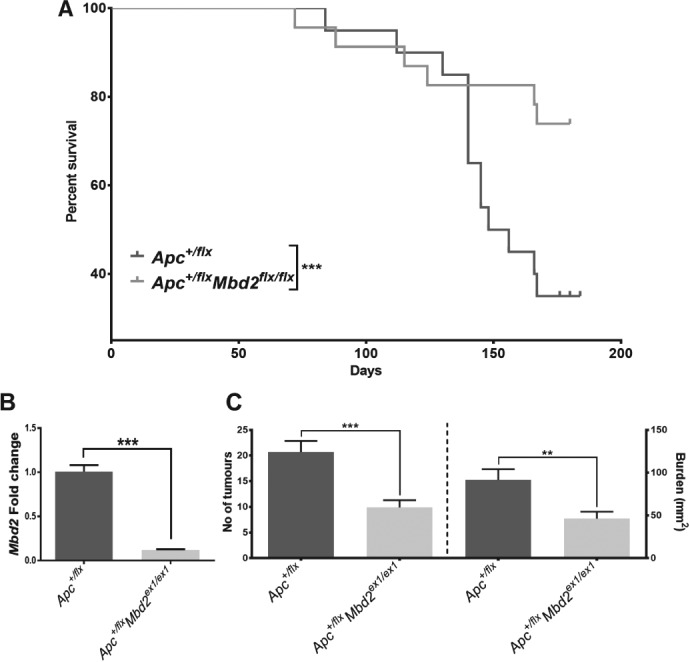
Mbd2 deficiency within the intestinal epithelia protects against Wnt‐driven tumourigenesis. (A) Kaplan–Meier survival curve indicating enhanced survival following epithelial loss of Ah‐creApc ^flx/flx^ Mbd2 ^ex1/ex1^ [N = 23, p = 0.005; log‐rank (Mantel–Cox) test] compared with Ah‐creApc ^flx/flx^ (N = 20). (B) RT‐qPCR expression analysis for Mbd2 expression 180 dpi after Ah‐cre induction indicates significant downregulation in Ah‐creApc ^flx/flx^ Mbd2 ^ex1/ex1^ mice (N = 4–6). (C) Analysis of tumour number (left panel) and burden (mm^2^; right panel) in surviving mice at 180 dpi indicates a significant decrease in the Ah‐creApc ^flx/flx^ Mbd2 ^ex1/ex1^ cohort.

Potentially, the intestinal tumour resistance of the *Mbd2* ^*−/−*^ mouse is due to an enhanced anti‐tumourigenic Th1/Ifng response, a loss of *Mbd2* epigenetic regulation within the intestinal epithelia or a combination of both. To clarify this situation, we utilised a conditional Cre‐Lox mouse to delete *Mbd2* solely within the intestinal epithelia, allowing us to establish the epithelial contribution of *Mbd2* to tumourigenesis. Mice carrying the *Ah‐cre* and *Mbd2* ^*ex1*^ transgenes were crossed to generate *Ah‐creMbd2* ^*ex1/ex1*^ mice, which following BNF induction deleted exon 1 of *Mbd2* specifically within the crypts of the intestinal epithelia. Cohorts of four to six mice were examined 4 days after deletion. In comparison to control cohorts, there was no alteration to the expression of Wnt target genes (*c‐Myc* and *Axin2*), the size of the crypt proliferative zone, Paneth cell localisation (Figure 1A–C) or genes characteristic of the differentiated cell types (supplementary material, Figure S1). To investigate the epithelial role of *Mbd2* in Wnt signalling, *Ah‐creMbd2* ^*ex1/ex1*^ mice were crossed to mice carrying an *Apc* ^*flx*^ allele, to generate *Ah‐creApc* ^*flx/flx*^ *Mbd2* ^*ex1/ex1*^ and control cohorts. Upon *Ah‐cre‐*driven *Apc* loss, we observed an upregulation of Wnt target genes, an increase in the size of the crypt proliferative zone, and mislocalisation of Paneth cells (Figure 1A–C), as we have previously reported 37. In comparison, the increase in expression of Wnt target genes *Axin2* (*p =* 0.0159) and *c‐Myc* (*p =* 0.0159), due to *Apc* deletion, was significantly attenuated by additional loss of *Mbd2* (*p =* 0.0159) (Figure 1A). This corresponded with a decrease in the size of the proliferative zone within the crypts of Leiberkühn (Figure 1B) and a partial rescue of the mislocalisation of Paneth cells (a characteristic of *Ah‐cre‐*driven *Apc* loss) (Figure 1C). These findings are consistent with our previously reported data using the *Ah‐creApc* ^*flx/flx*^ *Mbd2* ^*−/−*^ model 15, in which *Mbd2* is absent systemically. To investigate whether these changes would influence the initiation of intestinal tumourigenesis, cohorts of *Ah‐creApc* ^*+/flx*^ (*N* = 20) and *Ah‐creApc* ^*+/flx*^ *Mbd2* ^*ex1/ex1*^ (*N* = 23) mice were induced at 10–12 weeks of age and harvested at 180 days post‐induction (dpi). These mice are equivalent to the *Apc* ^*+/min*^ model, as they require the spontaneous loss of the remaining wild‐type allele for tumour initiation. The *Ah‐creApc* ^*+/flx*^ *Mbd2* ^*ex1/ex1*^ cohort showed a significant increase in survival compared with the *Ah‐creApc* ^*+/fl*^ control cohort (Figure 2A). Expression analysis on the whole intestine from six to eight animals within each cohort confirmed the continued absence of *Mbd2* within the epithelia (Figure 2B). At 180 dpi, the *Ah‐creApc* ^*+/flx*^ *Mbd2* ^*ex1/ex1*^ cohort had significantly fewer tumours and reduced burden compared with the control cohort (Figure 2C). In summary, the absence of *Mbd2* within the intestinal epithelia is well tolerated and sufficient to suppress Wnt signalling and tumourigenesis, replicating the phenotype observed in the *Apc* ^*+/min*^ *Mbd2* ^*−/−*^ mice and demonstrating a cell intrinsic mechanism for *Mbd2*. We next sought to investigate the role that *Mbd2* plays in intestinal inflammation.

### Mbd2 deficiency exacerbates DSS‐induced colitis

**Figure 3:**
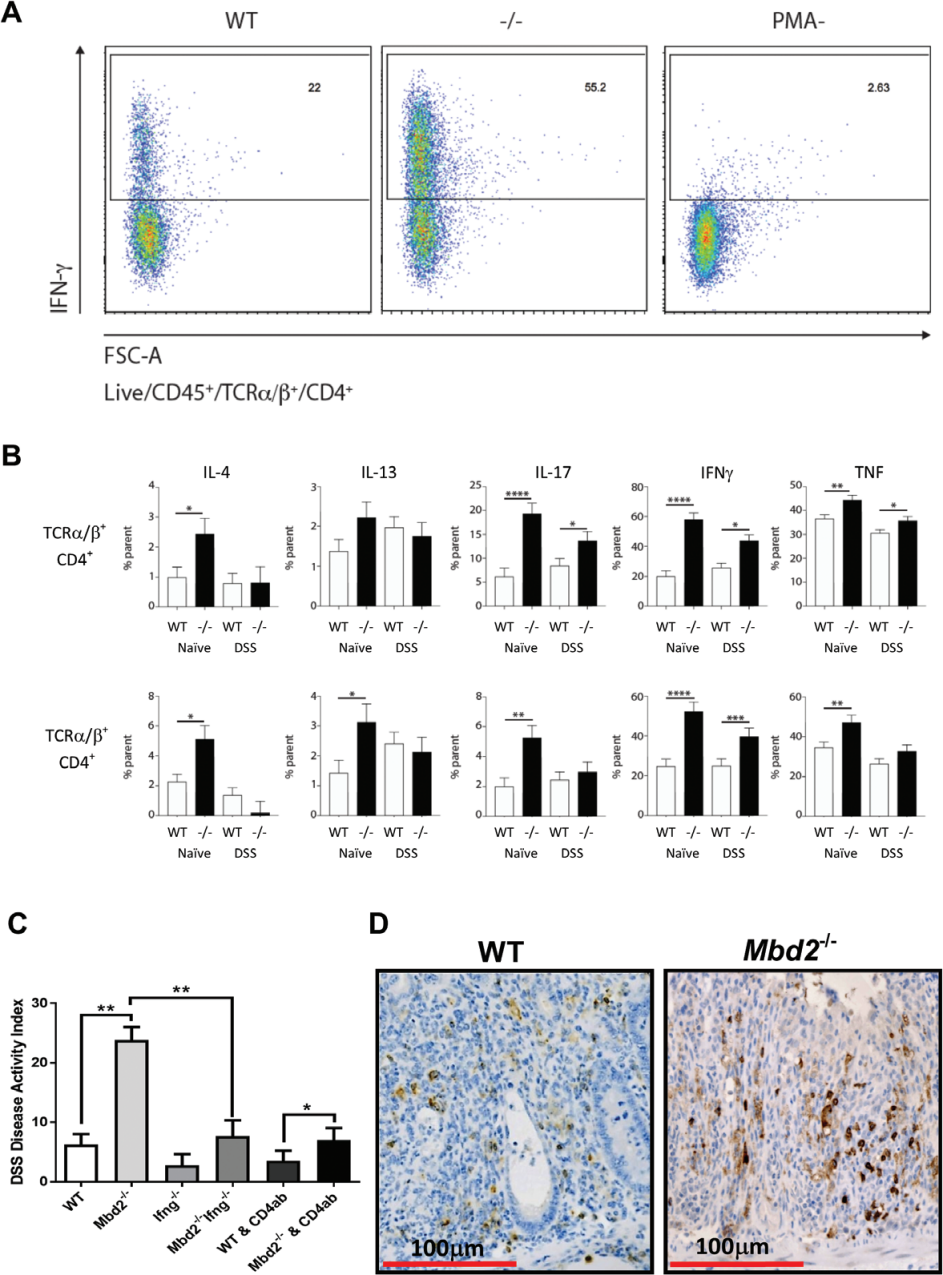
Mbd2 deficiency increases susceptibility to chronic intestinal inflammation. (A) Flow cytometry plots illustrating an increase in CD4^+^Ifng^+^ cells following PMA stimulation in Mbd2 ^−/−^ mice in comparison to WT. (B) Bar charts quantifying the cytokine expression profiles of CD4 and CD8 lamina propria lymphocytes as a percentage of the parent population in WT and Mbd2 ^−/−^ mice before and after DSS exposure. (C) Bar chart illustrating the DAI score 6 days after addition of 2% DSS to drinking water. (D) WT (left panel) and Mbd2 ^−/−^ (right panel) large intestine sections illustrating crypt loss and an increase in CD4^+^ cell number (brown) following DSS exposure.

As previous reports demonstrated that the *Mbd2* ^*−/−*^ mice have an enhanced CD4^+^ T‐helper type 1 response, characterised by an increase in *Ifng* levels, we first sought to verify this in our mice 8, 9. Lymphocytes were isolated from the large intestine lamina propria of wild type (WT) and *Mbd2* ^*−/−*^ mice and characterised using flow cytometry. The *Mbd2* ^*−/−*^ mice displayed a significant increase in the numbers of CD4^+^Ifng^+^ (Figure 3A) and CD8^+^cells expressing IL‐4, IL‐17, Ifng, and TNF, with CD8^+^ cells also displaying increased expression of IL‐13 (Figure 3A, B), similar to previously published data 8. The significantly elevated *Ifng* levels were further confirmed by an increase in mRNA expression and Ifng presence in the serum (supplementary material, Figure S2A, B). To investigate the impact of these changes on the large intestine, cohorts of ≥ 6 mice were administered 2% DSS (w/v) in drinking water *ad libitum* for 6 days to induce acute inflammation; experiments were repeated three times to assess reproducibility. The *Mbd2* ^*−/−*^ mouse demonstrated a significant increase in the disease activity index (DAI), histology score, weight loss, and large intestine atrophy compared with control mice (Figure 3C and supplementary material, Figure S2C–E). Analysis of lymphocytes from the lamina propria indicated an altered cytokine profile with a significant increase in CD4^+^ cells positive for IL‐17, Ifng, TNF, and CD8^+^Ifng^+^ cells (Figure 3B, D). As a key role for CD4^+^ cells and *Ifng* in DSS‐induced colitis has been previously shown, we repeated this experiment using *Ifng* ^*−/−*^ mice and neutralising CD4^+^ antibodies to determine their contribution to the phenotype. The absence of *Ifng* suppressed colitis, as previously shown 38, and significantly decreased severity in the *Mbd2* ^*−/−*^ *Ifng* ^*−/−*^ double‐knockout mice (Figure 3C). To address the importance of the CD4^+^ lymphocyte population, *Mbd2* ^*−/−*^ and control mice were administered a CD4 neutralising antibody −3, −1, and 0 days prior to induction of acute inflammation. The blockade of CD4^+^ cells reduced the DAI and weight loss scores to the levels previously observed by deleting *Ifng* (Figure 3C). To investigate whether the epithelial loss of *Mbd2* played a role in the altered immune response, we generated cohorts of *vil‐creER* ^*T2*^ *Mbd2* ^*ex1/ex1*^ to drive *Mbd2* deletion in the large intestinal epithelia. Four days after deletion of *Mbd2*, mice were exposed to 2% DSS in drinking water *ad libitum*. Six days following DSS exposure, the *vil‐creER* ^*T2*^ *Mbd2* ^*ex1/ex1*^ mice, in comparison to control mice, displayed a significant four‐fold decrease in *Mbd2* expression within the large intestine crypt epithelia but no difference in overall disease severity (supplementary material, Figure S3A, B). Further expression analysis within the large intestine indicated no alteration to genes characteristic of Th1 (*Ifng*), Th17 (*Tbx21*), and Tregs (*Foxp3*), which we previously demonstrated to be altered in the *Mbd2* ^*−/−*^ setting 8, 10, 12 (supplementary material, Figure S3C). In summary, the *Mbd2* ^*−/−*^ mouse is highly susceptible to DSS‐induced colitis, at least in part, due to the loss of *Mbd2* function specifically within the CD4^+^ cells and loss of appropriate regulation of the pro‐inflammatory cytokine Ifng. However, additional roles for *Mbd2* within the non‐epithelial cells of the stromal compartment cannot be discounted due to the epithelial specific nature of the *vil‐creER* ^*T2*^ model. We next sought to establish the role of this inflammation susceptibility in a cancer setting.

### DSS exposure leads to chronic mucosal colitis and tumourigenesis in the Mbd2 ^−/−^ mouse

**Figure 4:**
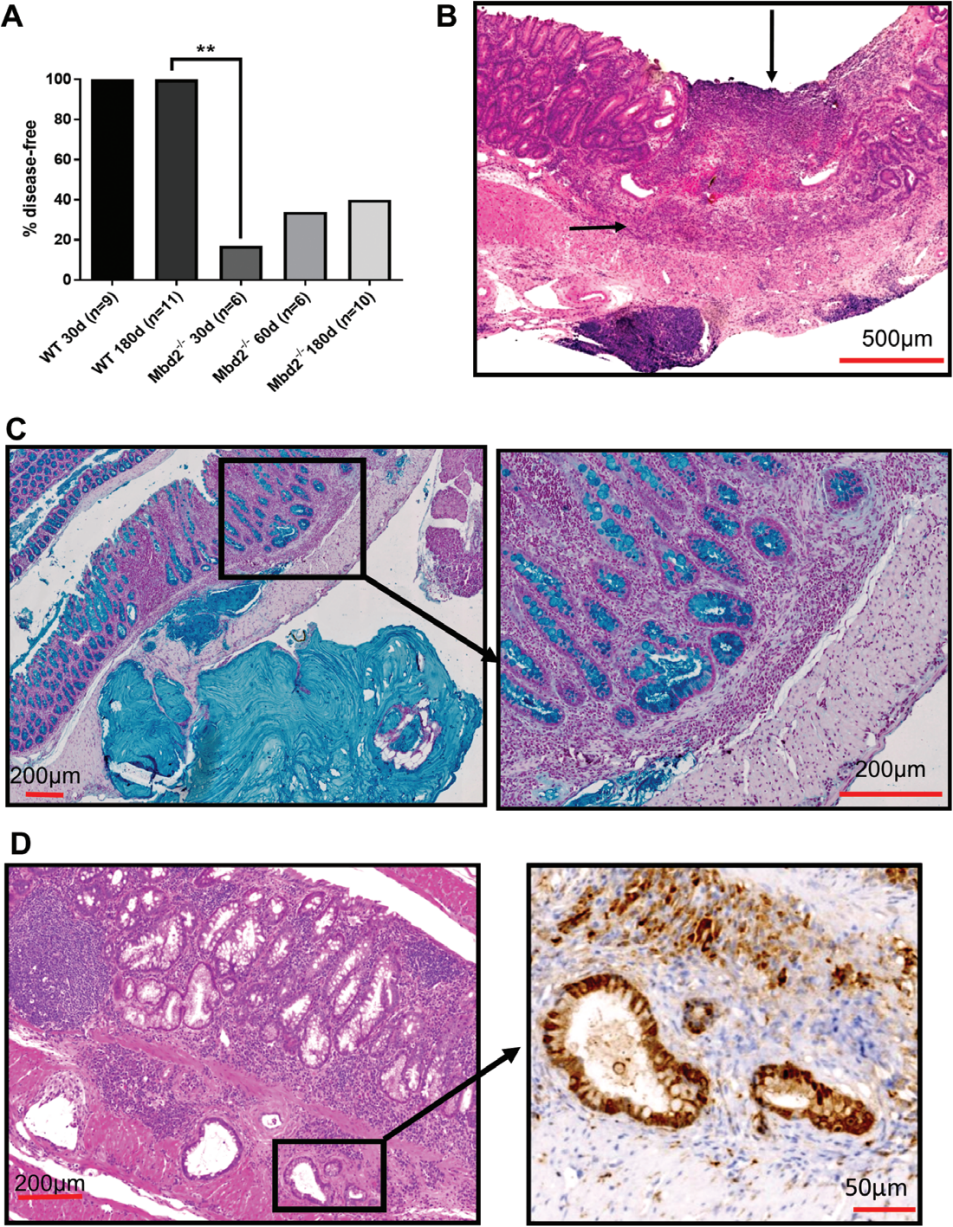
Mbd2 ^−/−^‐driven inflammation drives tumourigenesis in the large intestine. (A) Scoring for the presence of intestinal disease, following DSS withdrawal, indicating the percentage of mice with a histologically normal large intestine at different time points following exposure. d = days. (B) Representative image of Mbd2 ^−/−^ intestine 30 days post‐inflammation displaying a chronic mucosal colitis with superficial ulceration (↓) and mononuclear infiltrate (→). (C) Mbd2 ^−/−^ intestine 170 days post‐inflammation displaying an adenocarcinoma stained for mucin (blue). (D) Mbd2 ^−/−^ mucinous adenocarcinoma displaying heterogeneous nuclear β‐catenin (brown, →; inset).

To determine whether this increased susceptibility to colitis had a long‐term effect on intestinal health, cohorts of control and *Mbd2* ^*−/−*^ mice were administered 2% DSS for 6 days *ad libitum* and aged for 30, 60, and 170 days. The control *Mbd2* ^*+/+*^ littermates made a complete recovery following withdrawal of DSS and at 30 (*N* = 9) and 170 days (*N* = 11) post‐inflammation showed no sign of intestinal disease (Figure 4A). In contrast the *Mbd2* ^*−/−*^ mice failed to resolve the inflammation and developed a chronic mucosal colitis. At 30 days, 83% (5/6, *p =* 0.002) of *Mbd2* ^*−/−*^ mice showed continuing signs of colitis which included severe mucosal inflammation with severe diarrhoea, widespread crypt loss, superficial ulceration, focal active cryptitis with scattered crypt abscesses, and patches of epithelial regeneration (Figure 4B and supplementary material, Figure S4A). At 60 days, the severe diarrhoea and bleeding had subsided in all mice (*N* = 6); however, in the intestines of 66% (4/6) of these mice, an active chronic mucosal colitis remained, with a mononuclear cell infiltrate in the lamina propria, distortion of crypt architecture, regenerative epithelial hyperplasia, and crypt fission (supplementary material, Figure S4B). At 170 dpi, 60% (6/10, *p =* 0.0039) of the mice had flat lesions which were classified as mucinous adenocarcinoma (Figure 4C and supplementary material, Figure S4C). These lesions displayed nuclear β‐catenin staining indicating that they were driven via deregulation of the Wnt pathway (Figure 4D and supplementary material, Figure S4C). We have previously shown that absence of *Mbd2* in the epithelia alone is not sufficient to alter the acute immune response (supplementary material, Figure S3B). To confirm there was not a longer‐term phenotype, we generated cohorts of *vil‐creER* ^*T2*^ *Mbd2* ^*ex1/ex1*^ mice for analysis. At 180 days following exposure to 2% DSS in drinking water given *ad libitum*, these mice, in contrast to the systemic *Mbd2* ^*−/−*^ setting, showed no signs of intestinal disease (data not shown). These data suggest that the switch to a tumour‐promoting environment is dependent on loss of *Mbd2* in cells outside of the intestinal epithelia, with the cells of the immune system being the most likely candidates. We next sought to establish the effect of the *Mbd2* ^*−/−*^ inflammatory response on the tumour suppression observed in the *Apc* ^*+/min*^ *Mbd2* ^*−/−*^ intestine.

### Chronic inflammation overcomes Apc ^+/min^ Mbd2 ^−/−^ intestinal tumour suppression

**Figure 5:**
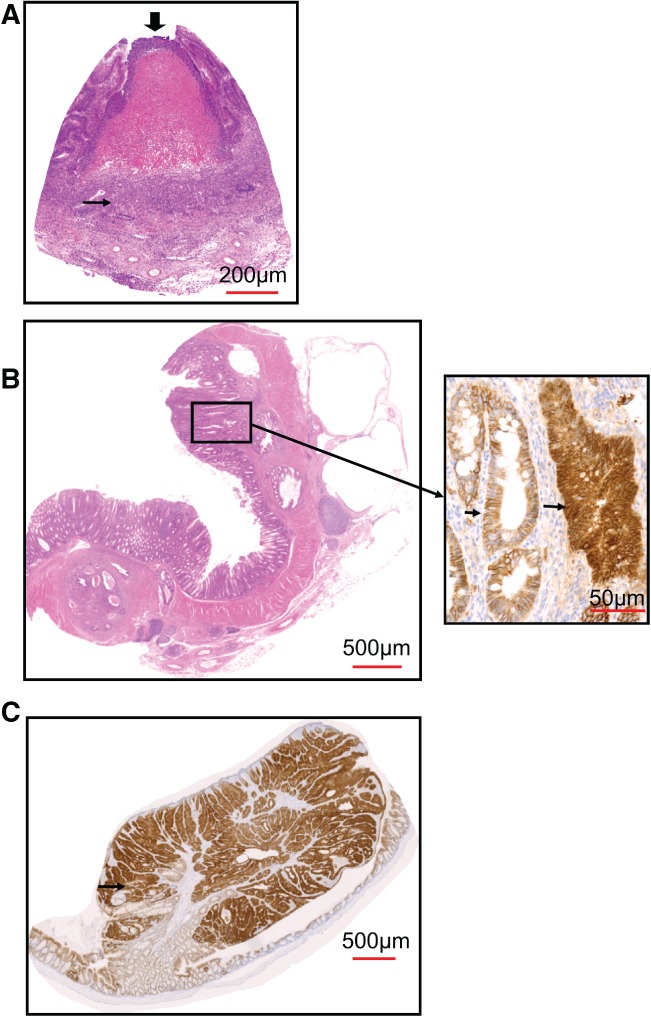
Inflammation overcomes the tumour resistance in the Apc ^+/min^ Mbd2 ^−/−^ intestine. (A) Representative image of Apc ^+/min^ Mbd2 ^−/−^ intestine 30 days post‐inflammation displaying a chronic mucosal colitis with large abscess (↓) and mononuclear infiltrate (→). (B) Apc ^+/min^ Mbd2 ^−/−^ intestine 180 days after onset of inflammation displaying a flat mucinous adenocarcinoma (left panel) with heterogeneous nuclear β‐catenin (→; right panel). (C) Adenoma from an Apc ^+/min^ large intestine 180 days post‐inflammation displaying mucin‐filled pockets (→) and extensive nuclear β‐catenin (brown).

As the *Apc* ^*+/min*^ *Mbd2* ^*−/−*^ is resistant to intestinal tumourigenesis, we addressed whether this is still the case following an acute inflammatory insult. *Apc* ^*+/min*^ *Mbd2* ^*−/−*^ mice at 10–12 weeks old were exposed to 2% DSS (w/v) for 6 days and allowed to age for 30 and 180 days. At 30 days after DSS withdrawal, all *Apc* ^*+/min*^ *Mbd2* ^*−/−*^ mice (4/4) still presented with chronic colitis (Figure 5A). At 180 days post‐DSS, 72% (8/11) of *Apc* ^*+/min*^ *Mbd2* ^*−/−*^ mice displayed mucinous adenocarcinoma with nuclear β‐catenin (Figure 5B), in contrast to control *Apc* ^*+/min*^ *Mbd2* ^*−/−*^ mice, which remained disease‐free at the same age, as reported previously 16. These lesions presented as flat tumours in contrast to the standard polypoid‐type lesions with extensive nuclear β‐catenin that developed in the *Apc* ^*+/min*^ model (Figure 5C). Thus, following the onset of a chronic inflammatory response, tumour suppression is lost in the *Apc* ^*+/min*^ *Mbd2* ^*−/−*^ large intestine; indicating that the protection afforded by the absence of *Mbd2* in the epithelia is overcome following the onset of an *Mbd2‐*deficient inflammatory response in the intestine.

### Loss of Mbd2 decreases the survival of Apc‐deficient stem cells

**Figure 6:**
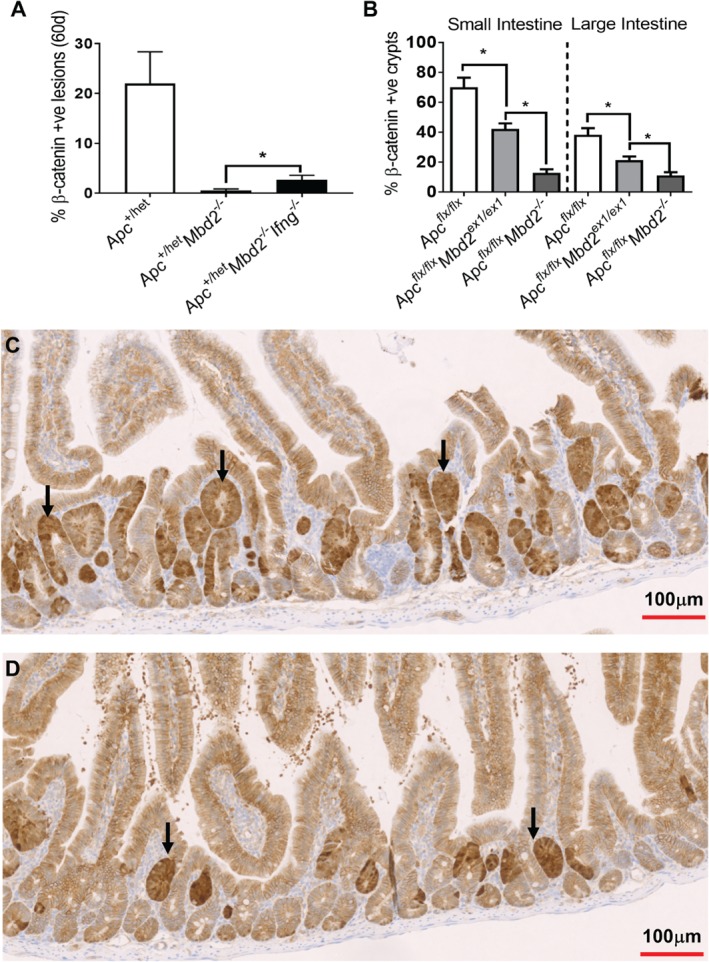
Mbd2 promotes the survival of Apc‐deficient stem cells. (A) Scoring of nuclear β‐catenin‐positive lesions indicates that at 60 days there is a reduction in Apc ^+/min^‐driven lesions due to Mbd2 deficiency which is partially dependent on Ifng (Apc ^+/min^, N = 6; Apc ^+/min^ Mbd2 ^−/−^, N = 6; and Apc ^+/min^ Mbd2 ^−/−^ Ifng ^−/−^, N = 6). (B) Following deletion of Apc in the ISC, using the Lgr5creER ^T2^ Apc ^flx/flx^ (N = 5) model, the number of nuclear β‐catenin‐positive crypts in the small (left panel) and large (right panel) intestine is significantly reduced in Lgr5creER ^T2^ Apc ^flx/flx^ Mbd2 ^flx/flx^ mice and further reduced in the Lgr5creER ^T2^ Apc ^flx/flx^ Mbd2 ^−/−^ (N = 4) setting. (C) Representative image of nuclear β‐catenin‐positive crypts (arrows; brown) in the small intestine 15 days after Lgr5creER ^T2^ Apc ^flx/flx^‐driven Apc deletion in ISCs and (D) in combination with Mbd2 ^ex1/ex1^ deletion.

Comparison of the data from our *Ah‐creApc* ^*+/flx*^ *Mbd2* ^*ex1/ex1*^ mice and our previously published *Ah‐creApc* ^*flx/flx*^ *Mbd2* ^*−/−*^ 15 and *Apc* ^*+/min*^ *Mbd2* ^*−/−*^ 16 mice indicated that the suppression of tumourigenesis, Wnt activation, and Paneth cell relocalisation phenotypes were enhanced in the *Mbd2* ^*−/−*^ mouse. These data suggested that in the *Mbd2* ^*−/−*^ model the epithelial and non‐epithelial phenotypes synergised to suppress tumourigenesis. As we have demonstrated a significant role for *Ifng* in the *Mbd2* ^*−/−*^ inflammatory response and it is a key player in inflammation and anti‐tumour immune responses 18, 20, 21, 23, 39, 40, we sought to assess the relevance of *Ifng* in the tumour suppression observed in *Apc* ^*+/min*^ *Mbd2* ^*−/−*^ mice. We generated *Apc* ^*+/min*^ *Mbd2* ^*−/−*^ *Ifng* ^*−/−*^ mice to explore whether loss of *Ifng* impacted on intestinal tumour suppression. As expected, at 60 days, the *Apc* ^*+/min*^ (*N* = 6) mice displayed significantly more nuclear β‐catenin‐positive lesions (average 22) in comparison to the *Apc* ^*+/min*^ *Mbd2* ^*−/−*^ mice (*N* = 6; average < 1) (Figure 6A). As we have previously demonstrated that epithelial loss of *Mbd2* alone is capable of intestinal tumour suppression (Figure 1), it is of note that the *Apc* ^*+/min*^ *Mbd2* ^*−/−*^ *Ifng* ^*−/−*^ mice (*N* = 6) displayed a small but significant increase in nuclear β‐catenin‐positive lesions (*p =* 0.03; average 2.6), indicating that in the absence of *Ifng* a small number of lesions can escape the tumour resistance conferred by epithelial *Mbd2* loss. In our previous work, where we investigated mice 4 days after acute *Apc* loss in the crypts of *Mbd2* ^*−/−*^ mice, this information may have been obscured 15. This acute *Apc* loss does not reflect the small number of mutated cells which initiate an individual tumour *in vivo* and presents over an insufficient time for an adaptive immunological anti‐tumorigenic phenotype to manifest. To overcome this, we targeted gene deletion to the intestinal stem cell (ISC) using the *Lgr5cre* transgene to generate *Lgr5creER* ^*T2*^ *Apc* ^*flx/flx*^ *Mbd2* ^*flx/flx*^ and *Lgr5creER* ^*T2*^ *Apc* ^*flx*/*flx*^ *Mbd2* ^*−/−*^ mice. These allowed us to delete *Apc* specifically within a proportion of ISCs, the cell of origin for CRC 41, allowing us to observe the effects of *Mbd2* deficiency on *Apc‐*deleted ISCs over a longer (15‐day) time frame. In comparison to the control *Lgr5creER* ^*T2*^ *Apc* ^*flx/flx*^ (*N* = 6) mice, we observed a significant reduction in the number of nuclear β‐catenin lesions in the *Lgr5creER* ^*T2*^ *Apc* ^*flx/flx*^ *Mbd2* ^*ex1/ex1*^ (*N* = 7) cohort, which was further reduced in the *Lgr5creER* ^*T2*^ *Apc* ^*+/flx*^ *Mbd2* ^*−/−*^ (*N* = 6) cohort (Figure 6B–D and supplementary material, Figure S5A–C). Potentially, the increased reduction of lesions in the *Mbd2* ^*−/−*^ mouse is due to synergy between the enhanced anti‐tumourigenic Th1/*Ifng* response and the loss of *Mbd2‐*mediated transcriptional silencing in the ISC. However, a role for other cells within the *Mbd2* ^*−/−*^ mouse cannot be excluded with this system.

### MBD2 loss is a rare event in colorectal cancer

Our data presented here and previously 15, 16 support the notion that in murine intestinal epithelium *Mbd2* is required to permit intestinal tumourigenesis. To investigate the relevance of *MBD2* in human intestinal cancer, we looked for evidence that it is preferentially retained in colorectal cancer, and whether its loss is associated with a positive prognosis. To achieve this, we examined *MBD2* expression in a panel of human intestinal tumours (TNM stage I–IV, *N* = 7 per stage). Expression analysis failed to detect any loss or alteration to the *MBD2* profile across the different tumour stages (supplementary material, Figure S6). We also examined publicly available colorectal cancer sequencing data using cBioPortal (http://www.cbioportal.org). *In silico* analysis indicated that the *MBD2* gene was classed as deep deleted in 6/2079 (0.28%) patients; while supportive of a role for *MBD2* in enabling tumourigenesis, the small number of samples in which *MBD2* was lost prevented any conclusive survival analysis.

## Discussion

Based on these preclinical data, the genes transcriptionally regulated by *Mbd2* within the immune system and stem cells from the normal and diseased intestinal epithelia offer a set of targets that play a key role in linking epigenetic changes to cancer. The finding that within the intestinal epithelia normal function of *Mbd2* is required to permit tumourigenesis is supported by clinical data; CRC sequencing data and a small study looking specifically at *MBD2* status 42 indicate that loss of *MBD2* is a rare event in CRC patients 43, 44. However, assigning a crucial role to a gene that requires no alteration to elicit a tumourigenic function is extremely difficult and emphasises the value of this type of preclinical research. Which of the *Mbd2* regulated genes within the intestinal epithelia are responsible for this protection remains to be established. However, it is now clear that the broad role of *Mbd2* in the immune system influences *Apc*‐deficient epithelial stem cells. Evidence is now emerging that cross talk between Th cells and MHC II‐expressing ISCs regulates ISC numbers and differentiation status 45. Recent work has demonstrated a key role for two inflammatory cytokines, Ifng and Tnf, and the JAK/STAT‐1 signalling pathway in the reserve ISC regenerative response to acute intestinal inflammation 46. Thus, the role of *Mbd2* in controlling the CD4 and Ifng immune response is likely to be of great interest because ‘tumour‐promoting inflammation’ is now identified as an enabling characteristic in the hallmarks of cancer 47. Further investigation of *Mbd2* functions should aid at understanding the links between a type 1 acute inflammatory response (the DSS‐induced colitis model; Figure 2A) and an anti‐tumour response. Potentially, in the early stages, the acute Th1–Ifng inflammatory response in the *Mbd2* ^*−/−*^ mouse is associated with tumour clearance. In humans, a Th1–Ifng response is a characteristic of cancer immune surveillance 48, associated with Th1 CD4^+^ and CD8^+^ T cells which directly regulate tumour cell cytotoxicity or induce senescence, while indirectly polarizing innate immune cells towards tumour suppression 20, 49. However, the inability of these *Mbd2* ^*−/−*^ mice to resolve the inflammation, resulting in chronically inflamed intestines, despite irritant withdrawal, suggests either an auto‐immune response to a self‐antigen, a neo‐antigen generated by the altered transcriptional profile 50, or a response to bacterial translocation as a result of impaired epithelia integrity 51. This chronic pro‐tumourigenic inflammatory environment is akin to the inflammation‐associated cancer that develops in Crohn's and other colitis patients. In our mouse model, the inflammation can override the *Mbd2‐*deficient‐dependent anti‐tumour suppression mechanism to such an extent that *Apc* ^*+/min*^ *Mbd2* ^*−/−*^ mice develop adenocarcinomas, which are rarely, if ever, seen in this model without an inflammatory insult. This loss of inflammatory control is potentially due to the known role of Mbd2 in promoting T‐reg cell function 10. These cells suppress the immune responses of other cells and maintain self‐tolerance; experimental depletion of these cells in animal experiments leads to colitis, whereas in CRC patients their accumulation is associated with progression 52. Thus, Mbd2 function may play a significant role in the progression from a protective acute response to a chronic tumour‐promoting environment.

This work highlights the role that epigenetics plays in the cells of a tumour and its environment. These changes can affect the tumour itself and the delicate balance between acute and chronic inflammation which elicits anti‐ or pro‐tumourigenic effects. Taking into consideration that in excess of 95% of CRC cases are sporadic, arising in individuals with no identified genetic predisposition 53, demonstrates that the aetiology of CRC is multifactorially linked to genetic mutations, diet, inflammatory processes, ageing, and, more recently, the gut microbiota. The study of the epigenetic mechanisms which underpin these gene–environment interactions is crucial to understand how to prevent and control this disease. Epigenetic regulation, via DNA methylation, is commonly used by all normal cells to ensure proper regulation of gene expression and stable gene silencing, and is invariably altered in tumourigenesis. Recent technological advances are now leading to the identification of new genes and loci associated with inflammatory bowel diseases and CRC, based on generating methylome maps 54, 55. However, concentrating solely on the DNA methylome within the intestinal epithelium neglects the fact that the majority of the CRC‐associated factors impact on the epigenome within the entire body and not just the intestinal epithelium. Ultimately, interpreting the DNA methylation changes will require a more holistic approach to explain the links between the environment and CRC. We can begin to move towards this goal by exploiting existing knowledge of how DNA methylation is interpreted. The myriad of DNA methylation differences observed at an organism level are interpreted by a relatively small number of proteins 4. Given the small number of proteins capable of interpreting the DNA methylation signal, it stands to reason that some will play a major role in the multiple changes that occur to permit tumour initiation and progression. In this study, we have demonstrated how loss of a single gene involved in the interpretation of DNA methylation has pleiotropic effects on inflammation and cancer that could be environmentally regulated. In the context of understanding the relationship between the multiple factors associated with CRC, this emphasises that non‐cell intrinsic epigenetic changes beyond the target tissue should be considered in the attempt to unravel mechanisms.

## Author contributions statement

LP designed the research. LP, SM, TP, KG, AB (Cardiff), CT, NB‐L, GRJ, PCC, ASM, and GW performed research. HO and AG provided reagents and critical analysis of the manuscript. LP, MS, TP, AB, ARC, and OS analysed data and provided critical analysis of the manuscript. LP drafted the manuscript.

**SUPPLEMENTARY MATERIAL ONLINE**

**Supplementary figure legends**

**Figure S1.** Expression analysis of intestine 4 days after epithelial *Mbd2* deletion indicates that cell homeostasis is maintained

**Figure S2.** Deficiency of *Mbd2* increases *Ifng* levels and enhances DSS‐induced colitis

**Figure S3.** Intestinal DSS response is unaltered following *vil‐creER* ^*T2*^‐driven epithelial loss of *Mbd2*

**Figure S4.** Following an acute inflammatory insult, the *Mbd2*‐deficient intestine develops chronic mucosal colitis (6 days post‐DSS administration)

**Figure S5.** *Mbd2* promotes the survival of *Apc‐*deficient stem cells

**Figure S6.** *MBD2* expression is constant irrespective of intestinal tumour stage

## Supporting information


**Supplementary figure legends**

**Figure S1.** Expression analysis of intestine 4 days after epithelial Mbd2 deletion indicates that cell homeostasis is maintained. RT‐qPCR results indicate no significant alteration in the expression of genes representing the stem cells (Lgr5) or enterocyte (Sis), enteroendocrine (Syp), goblet (Muc2), and Paneth cell lineages (Lyz1) (p > 0.05).

**Figure S2.** Deficiency of Mbd2 increases Ifng levels and enhances DSS‐induced colitis. (A) Expression analysis for Ifng in whole mouse intestine using RT‐qPCR indicated an approximate 30‐fold increase (N = 4–6, p = 0.0159; a decrease in dCt levels indicates upregulation). (B) A cytokine bead array assay showed a significant increase in Ifng serum levels in Mbd2 ^−/−^ mice (N = 4, p = 0.0159). Following 6 days' exposure to 2% DSS in drinking water, scoring indicated a significant increase in the histopathology score (C) and weight loss (D) (expressed as a percentage of the starting weight) in the Mbd2 ^−/−^ setting which was attenuated by the loss of Ifng. (E) Representative images indicating colon atrophy in starting‐weight‐matched mice following exposure to 2% DSS in drinking water.

**Figure S3.** Intestinal response to DSS is unaltered following vil‐creER ^T2^‐driven epithelial loss of Mbd2. (A) RT‐qPCR results for Mbd2 expression 6 days after its deletion in the intestinal epithelia indicate a significant downregulation of Mbd2 (N = 4–6). (B) Disease activity index (DAI) scores indicated no significant change of the severity of 2% DSS exposure following loss of epithelial Mbd2 in comparison to WT and Mbd2 ^−/−^ intestines. (C) Following epithelial loss of Mbd2 and exposure to 2% DSS, the expression of genes representative of Treg (Foxp3), Th1 (Ifng), and Th17 (Tbx21) is unaltered in the large intestine.

**Figure S4.** Following an acute inflammatory insult, the Mbd2‐deficient intestine develops chronic mucosal colitis (6 days post‐DSS administration). (A) Representative H&E picture of Mbd2 ^−/−^ intestine (A) at 30 days, with a crypt abscess (↓) and widespread mononuclear infiltration of the lamina propria; (B) at 60 days, crypt fission (→); and (C) at 180 days, an adenocarcinoma with nuclear β‐catenin (brown ↓, inset). Images were excised from scans taken using Zeiss Axioscan Zen software.

**Figure S5.** Mbd2 promotes the survival of Apc‐deficient stem cells. Immunostaining for β‐catenin (brown) in sections of small intestine from (A) Lgr5creER ^T2^ Apc ^flx/flx^, (B) Lgr5creER ^T2^ Apc ^flx/flx^ Mbd2 ^ex1/ex1^, and (C) Lgr5creER ^T2^ Apc ^flx/flx^ Mbd2 ^−/−^ mice at 15 days following tamoxifen induction. A reduction in nuclear β‐catenin lesions (arrowhead, dark brown areas) is seen because of epithelial Mbd2 loss and further reduction in the Mbd2 ^−/−^ setting.

**Figure S6.** MBD2 expression is constant irrespective of intestinal tumour stage. qRT‐PCR data indicating that MBD2 expression is consistent across UICC stage I–IV tumours (N = 7 per group).
