# Supplementary material for: Mbd2 enables tumourigenesis within the intestine while preventing tumour‐promoting inflammation
Source: J Pathol. 2018 May 16;245(3):270–82. doi: 10.1002/path.5074 (PMC6032908; doi:10.1002/path.5074)
Supplement: Supplementary file 1 — Supplementary figure legends [file PATH-245-270-s007.docx]

**Supplementary figure legends**

**Figure S1.** Expression analysis of intestine 4 days after epithelial *Mbd2* deletion indicates that cell homeostasis is maintained. RT-qPCR results indicate no significant alteration in the expression of genes representing the stem cells (*Lgr5*) or enterocyte (*Sis*), enteroendocrine (*Syp*), goblet (*Muc2*), and Paneth cell lineages (*Lyz1*) (*p* > 0.05).

**Figure S2.** Deficiency of *Mbd2* increases *Ifng* levels and enhances DSS-induced colitis. (A) Expression analysis for *Ifng* in whole mouse intestine using RT-qPCR indicated an approximate 30-fold increase (*N* = 4–6, *p* = 0.0159; a decrease in dCt levels indicates upregulation). (B) A cytokine bead array assay showed a significant increase in Ifng serum levels in *Mbd2^−/−^* mice (*N* = 4, *p* = 0.0159). Following 6 days’ exposure to 2% DSS in drinking water, scoring indicated a significant increase in the histopathology score (C) and weight loss (D) (expressed as a percentage of the starting weight) in the *Mbd2^−/−^* setting which was attenuated by the loss of *Ifng*. (E) Representative images indicating colon atrophy in starting-weight-matched mice following exposure to 2% DSS in drinking water.

**Figure S3.** Intestinal response to DSS is unaltered following *vil-creER^T2^*-driven epithelial loss of *Mbd2*. (A) RT-qPCR results for *Mbd2* expression 6 days after its deletion in the intestinal epithelia indicate a significant downregulation of *Mbd2* (*N* = 4–6). (B) Disease activity index (DAI) scores indicated no significant change of the severity of 2% DSS exposure following loss of epithelial *Mbd2* in comparison to WT and *Mbd2^−/−^* intestines. (C) Following epithelial loss of *Mbd2* and exposure to 2% DSS, the expression of genes representative of Treg (*Foxp3*), Th1 (*Ifng*), and Th17 (*Tbx21*) is unaltered in the large intestine.

**Figure S4.** Following an acute inflammatory insult, the *Mbd2*-deficient intestine develops chronic mucosal colitis (6 days post-DSS administration). (A) Representative H&E picture of *Mbd2^−/−^* intestine (A) at 30 days, with a crypt abscess (↓) and widespread mononuclear infiltration of the lamina propria; (B) at 60 days, crypt fission (→); and (C) at 180 days, an adenocarcinoma with nuclear β-catenin (brown ↓, inset). Images were excised from scans taken using Zeiss Axioscan Zen software.

**Figure S5.** *Mbd2* promotes the survival of *Apc-*deficient stem cells. Immunostaining for β-catenin (brown) in sections of small intestine from (A) *Lgr5creER^T2^Apc^flx/flx^*, (B) *Lgr5creER^T2^Apc^flx/flx^Mbd2^ex1/ex1^*, and (C) *Lgr5creER^T2^Apc^flx/flx^Mbd2^−/−^* mice at 15 days following tamoxifen induction. A reduction in nuclear β-catenin lesions (arrowhead, dark brown areas) is seen because of epithelial *Mbd2* loss and further reduction in the *Mbd2^−/−^* setting.

**Figure S6.** *MBD2* expression is constant irrespective of intestinal tumour stage. qRT-PCR data indicating that *MBD2* expression is consistent across UICC stage I–IV tumours (*N* = 7 per group).
