# Supplementary figures and images for: Mbd2 enables tumourigenesis within the intestine while preventing tumour‐promoting inflammation
Source: J Pathol. 2018 May 16;245(3):270–82. doi: 10.1002/path.5074 (PMC6032908; doi:10.1002/path.5074)

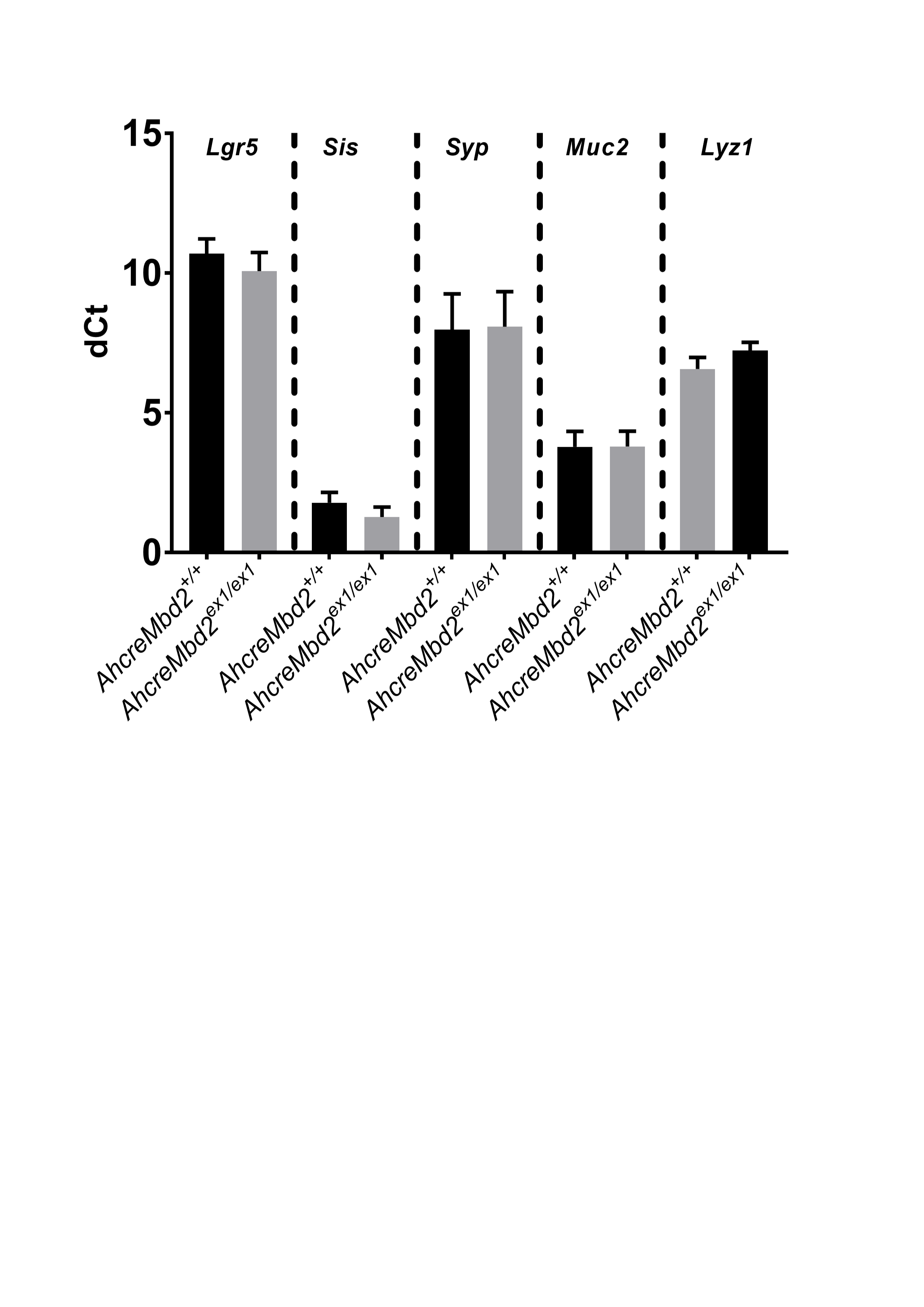

Supplement: Supplementary file 2 — Figure S1. Expression analysis of intestine 4 days after epithelial Mbd2 deletion indicates that cell homeostasis is maintained. RT‐qPCR results indicate no significant alteration in the expression of genes representing the stem cells (Lgr5) or enterocyte (Sis), enteroendocrine (Syp), goblet (Muc2), and Paneth cell lineages (Lyz1) (p > 0.05). [file PATH-245-270-s001.tif]

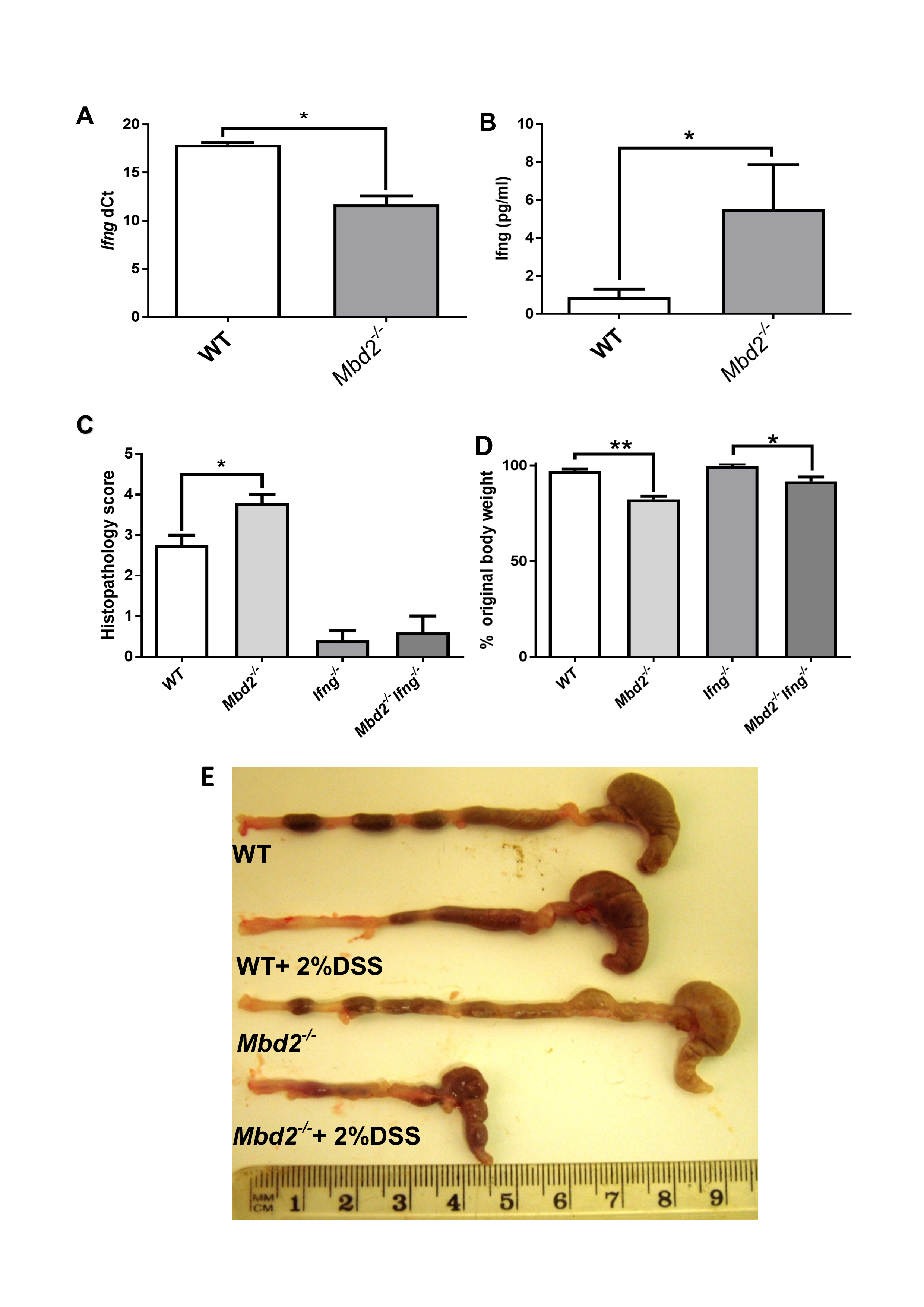

Supplement: Supplementary file 3 — Figure S2. Deficiency of Mbd2 increases Ifng levels and enhances DSS‐induced colitis. (A) Expression analysis for Ifng in whole mouse intestine using RT‐qPCR indicated an approximate 30‐fold increase (N = 4–6, p = 0.0159; a decrease in dCt levels indicates upregulation). (B) A cytokine bead array assay showed a significant increase in Ifng serum levels in Mbd2 −/− mice (N = 4, p = 0.0159). Following 6 days' exposure to 2% DSS in drinking water, scoring indicated a significant increase in the histopathology score (C) and weight loss (D) (expressed as a percentage of the starting weight) in the Mbd2 −/− setting which was attenuated by the loss of Ifng. (E) Representative images indicating colon atrophy in starting‐weight‐matched mice following exposure to 2% DSS in drinking water. [file PATH-245-270-s002.tif]

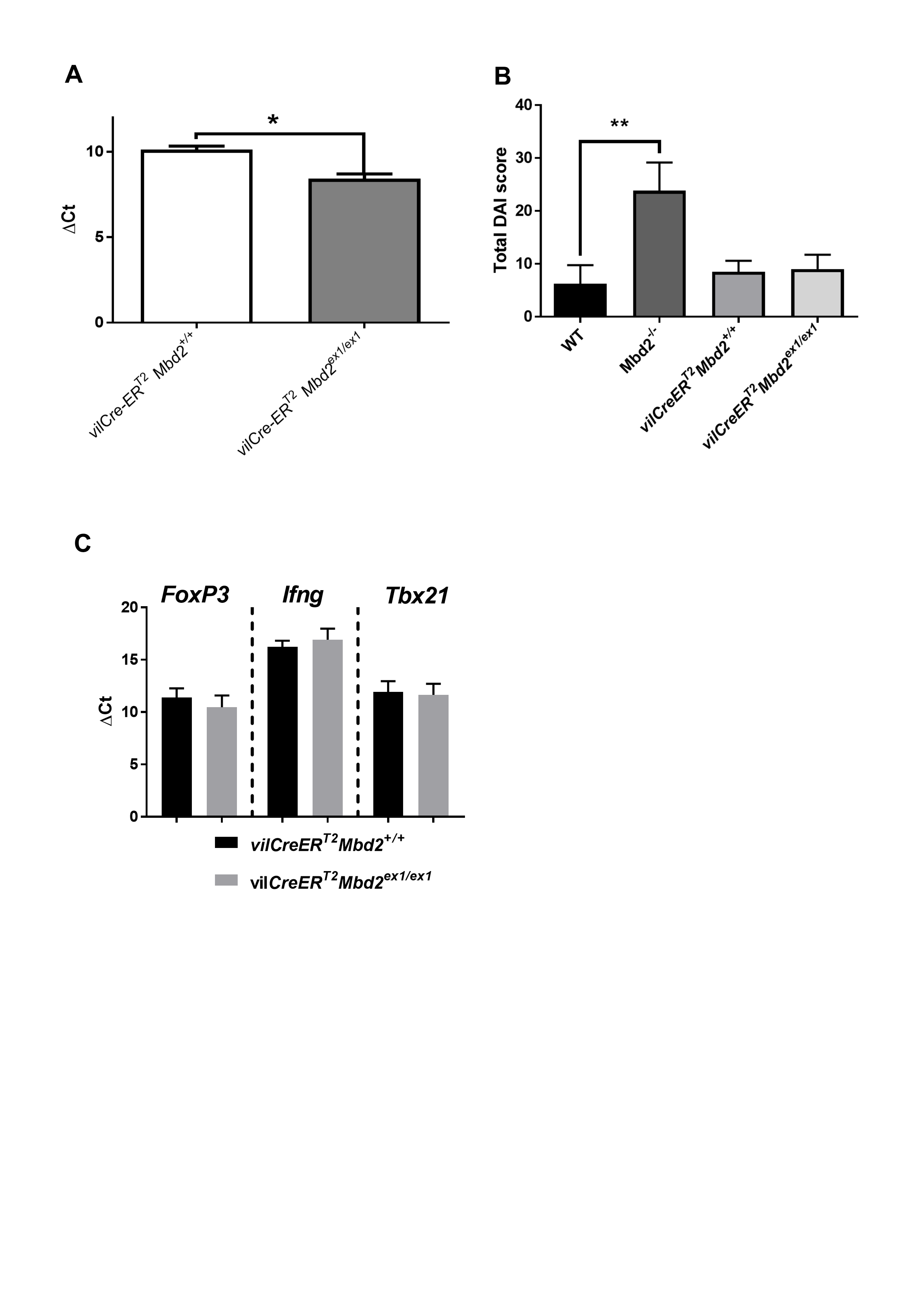

Supplement: Supplementary file 4 — Figure S3. Intestinal response to DSS is unaltered following vil‐creER T2‐driven epithelial loss of Mbd2. (A) RT‐qPCR results for Mbd2 expression 6 days after its deletion in the intestinal epithelia indicate a significant downregulation of Mbd2 (N = 4–6). (B) Disease activity index (DAI) scores indicated no significant change of the severity of 2% DSS exposure following loss of epithelial Mbd2 in comparison to WT and Mbd2 −/− intestines. (C) Following epithelial loss of Mbd2 and exposure to 2% DSS, the expression of genes representative of Treg (Foxp3), Th1 (Ifng), and Th17 (Tbx21) is unaltered in the large intestine. [file PATH-245-270-s003.tif]

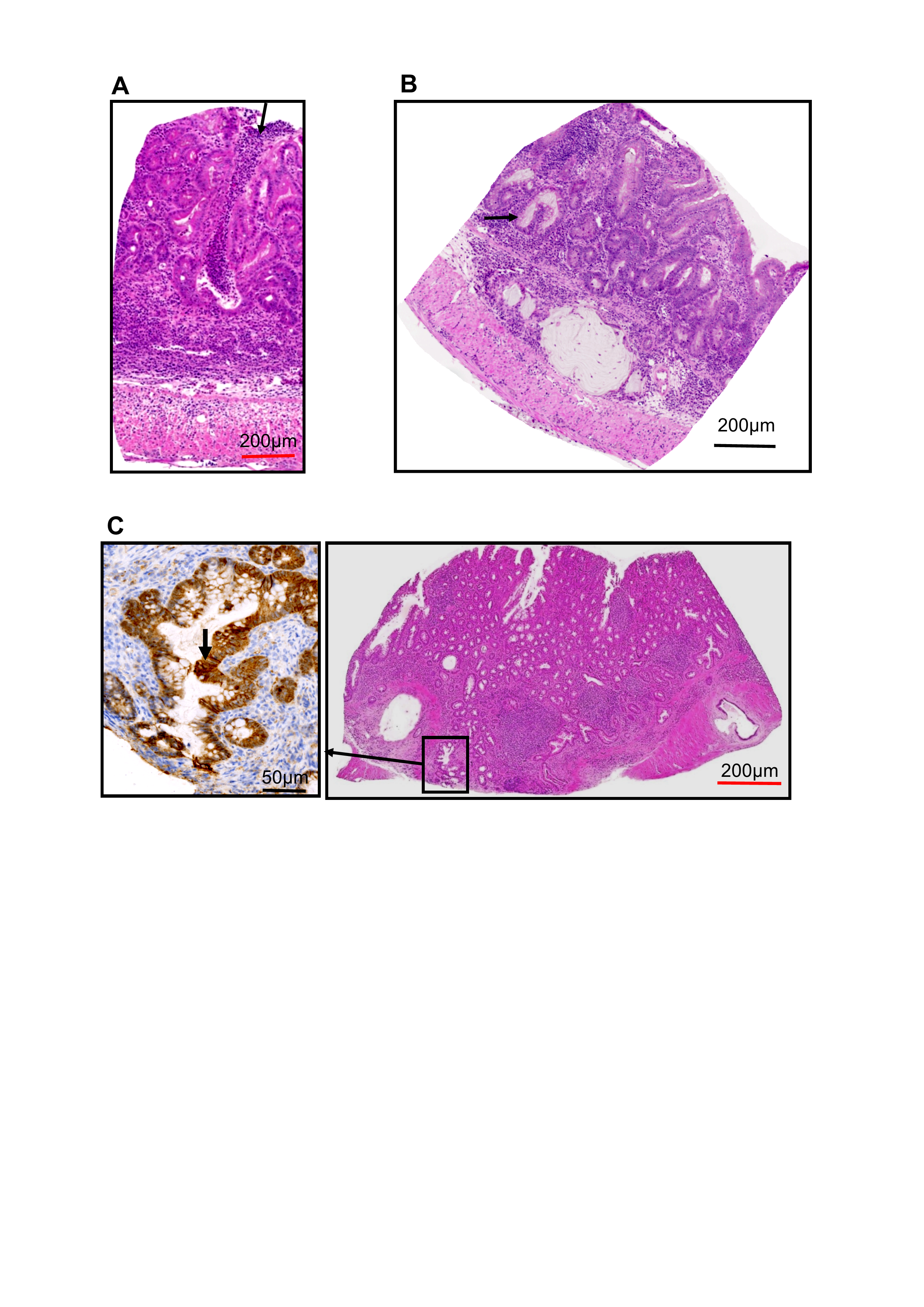

Supplement: Supplementary file 5 — Figure S4. Following an acute inflammatory insult, the Mbd2‐deficient intestine develops chronic mucosal colitis (6 days post‐DSS administration). (A) Representative H&E picture of Mbd2 −/− intestine (A) at 30 days, with a crypt abscess (↓) and widespread mononuclear infiltration of the lamina propria; (B) at 60 days, crypt fission (→); and (C) at 180 days, an adenocarcinoma with nuclear β‐catenin (brown ↓, inset). Images were excised from scans taken using Zeiss Axioscan Zen software. [file PATH-245-270-s004.tif]

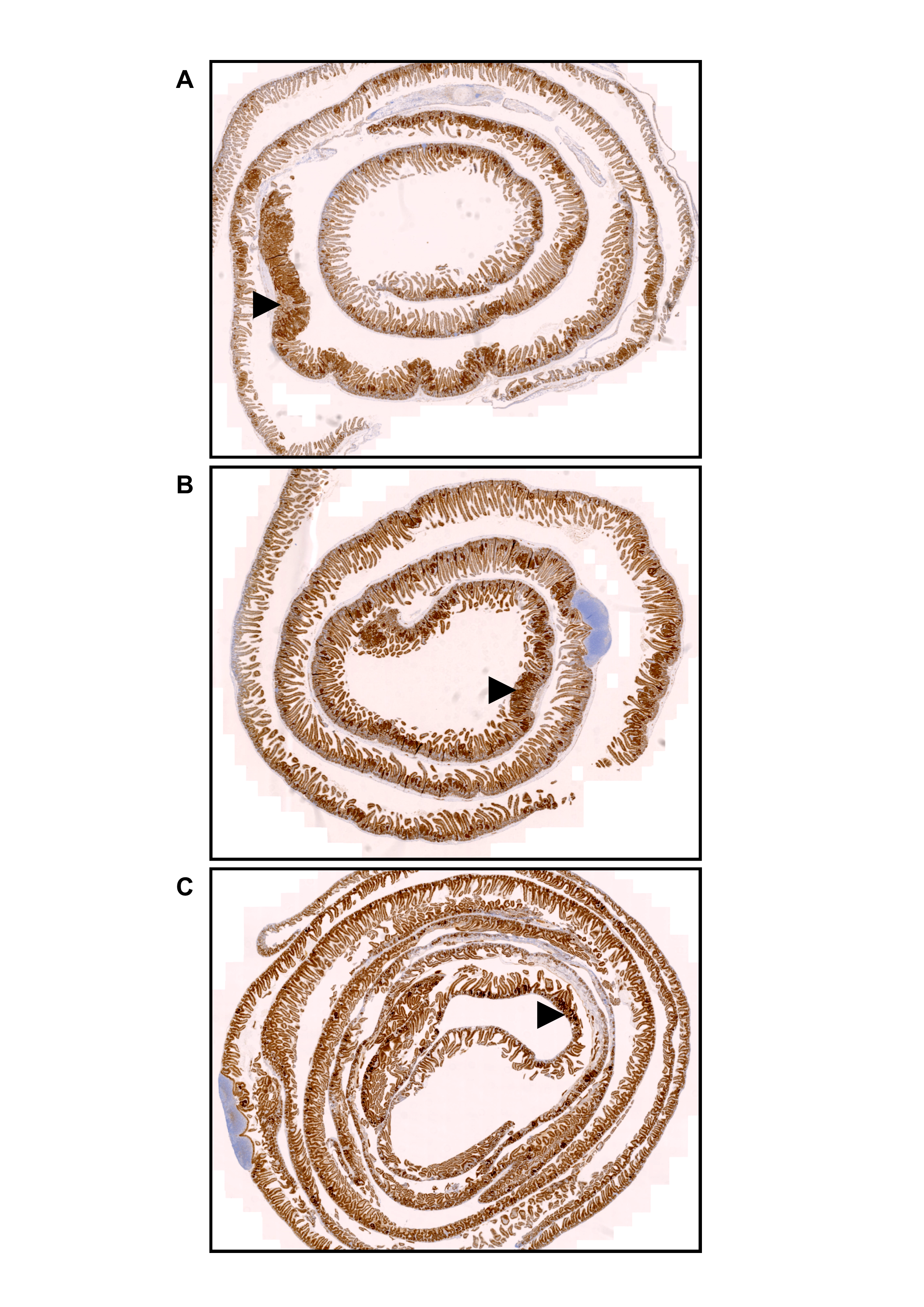

Supplement: Supplementary file 6 — Figure S5. Mbd2 promotes the survival of Apc‐deficient stem cells. Immunostaining for β‐catenin (brown) in sections of small intestine from (A) Lgr5creER T2 Apc flx/flx, (B) Lgr5creER T2 Apc flx/flx Mbd2 ex1/ex1, and (C) Lgr5creER T2 Apc flx/flx Mbd2 −/− mice at 15 days following tamoxifen induction. A reduction in nuclear β‐catenin lesions (arrowhead, dark brown areas) is seen because of epithelial Mbd2 loss and further reduction in the Mbd2 −/− setting. [file PATH-245-270-s005.tif]

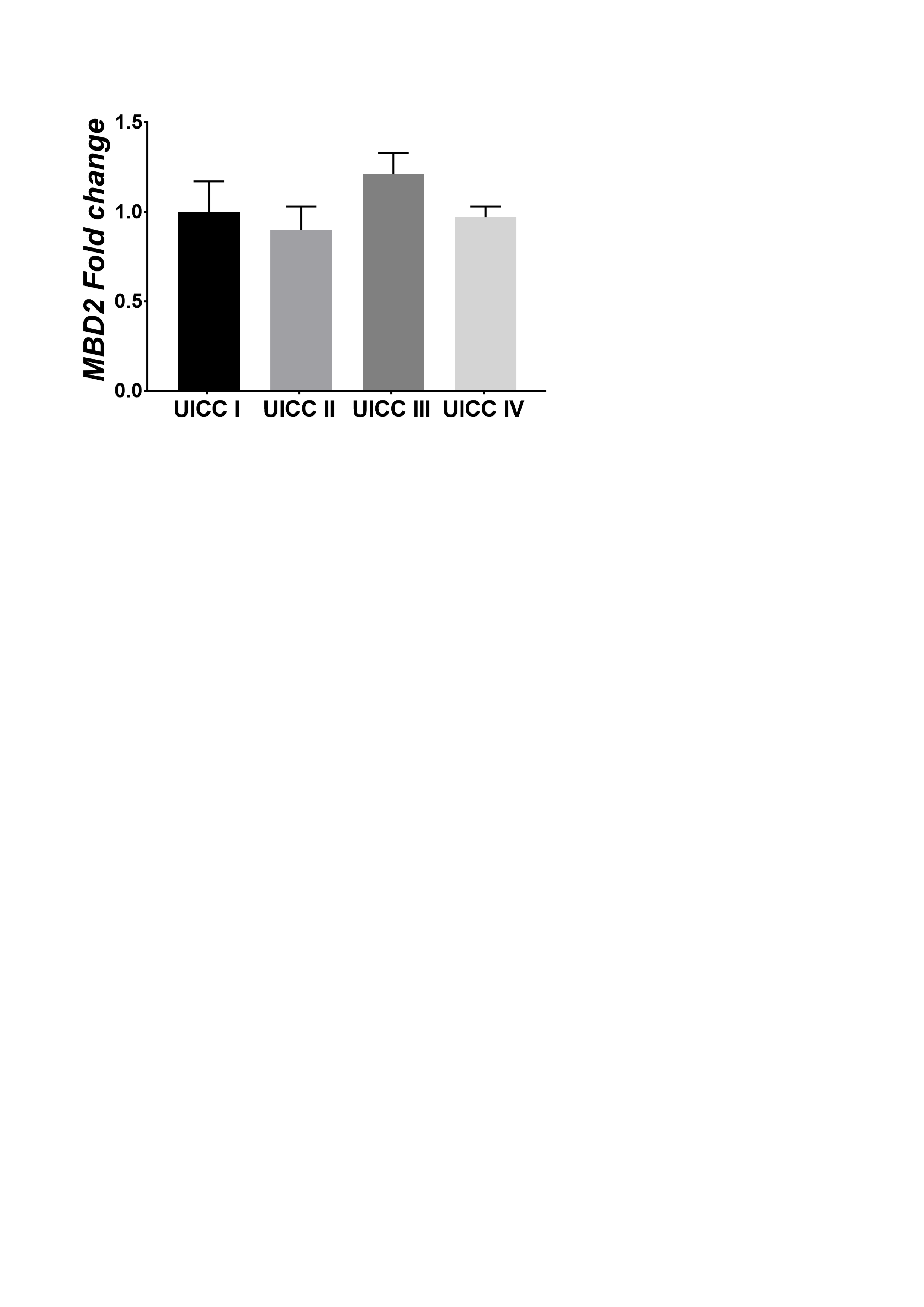

Supplement: Supplementary file 7 — Figure S6. MBD2 expression is constant irrespective of intestinal tumour stage. qRT‐PCR data indicating that MBD2 expression is consistent across UICC stage I–IV tumours (N = 7 per group). [file PATH-245-270-s006.tif]
